# Germination and Outgrowth of *Bacillus subtilis* Spores Deficient in BER and DisA Unveil Alternative Genetic Checkpoints

**DOI:** 10.3390/microorganisms13040939

**Published:** 2025-04-18

**Authors:** Alejandra Rangel-Mendoza, Luz I. Valenzuela-García, Eduardo A. Robleto, Mario Pedraza-Reyes

**Affiliations:** 1 Department of Biology, University of Guanajuato, Guanajuato 36050, Guanajuato, Mexico; a.rangelmendoza@ugto.mx; 2 Department of Sustainable Engineering, Advanced Materials Research Center (CIMAV), Subsede-Durango, Durango 34147, Durango, Mexico; luz.valenzuela@cimav.edu.mx; 3 School of Life Sciences, University of Nevada, Las Vegas, NV 89154, USA; eduardo.robleto@unlv.edu

**Keywords:** *Bacillus subtilis*, BER, checkpoint, DisA, germination/outgrowth

## Abstract

During *Bacillus subtilis* spore germination/outgrowth, the rehydration of the spore core and activation of aerobic metabolism can generate reactive oxygen species (ROS)-promoted DNA lesions that are repaired via the base excision repair pathway (BER). Accordingly, spores deficient in the AP-endonucleases (APEs) Nfo and ExoA exhibit a delayed outgrowth that is suppressed following disruption of the checkpoint protein DisA. Here, we report that DisA-independent DNA damage checkpoints operate during *B. subtilis* spore outgrowth. Consistent with this notion, spores lacking Nfo, ExoA, and Nth, which functions as an APE, did not suppress delayed outgrowth following *disA* disruption. Furthermore, in reference to the ∆*nfo* ∆*exoA* ∆*nth* spores, spores deficient for these APEs and DisA displayed a significantly higher number of oxidative genetic lesions and failed to properly segregate its chromosome during the first round of replication in the outgrowth stage. Finally, we found that DisA promotes low-fidelity repair and replication events, as revealed by DNA-alkaline gel electrophoresis (AGE) as well as spontaneous and H_2_O_2_-promoted Rif^R^ mutagenesis. Overall, our results unveil the existence of DisA-independent DNA damage checkpoint(s) that are activated by genomic lesions of an oxidative nature during spore germination and outgrowth, ensuring a proper transition to vegetative growth.

## 1. Introduction

The Gram-positive bacterium *Bacillus subtilis* has developed multiple strategies to contend with environmental conditions inappropriate for growth, establishing, during the stationary phase of growth, cell subpopulations with distinct morphophysiological properties including competence and sporogenesis [1]. Sporulation is regulated by a cascade of sigma (σ) factors and begins with the phosphorylation of Spo0A, which triggers asymmetric cell division, resulting in the formation of two unequally sized compartments: the forespore and the mother cell [2,3]. Subsequently, the forespore is engulfed by the mother cell, inducing transcriptional changes that lead to the formation of an external protective layer around the forespore. Upon completion of its maturation, a highly resistant spore is released [3]. *B. subtilis* spores can remain dormant for undefined periods until environmental conditions are appropriate for returning to vegetative growth through a process called germination/outgrowth [1,4]. Germination is initiated by the detection of nutrients through receptors located in the inner membrane of the spore, triggering an irreversible sequence of events that includes: (i) the release of monovalent and divalent cations (H^+^, Zn^2+^, and Ca^2+^); (ii) the expulsion of dipicolinic acid (DPA); (iii) core hydration; and (iv) cortex peptidoglycan hydrolysis [5]. These steps enable the resumption of metabolism and macromolecule synthesis, leading to the emergence of a vegetative cell (outgrowth) [6]. Prokaryotes regulate cell differentiation processes through a wide diversity of checkpoints [7]. In *Caulobacter crescentus*, CtrA controls DNA replication and cell division through phosphorylation and proteolysis, while in *Escherichia coli*, SulA functions as a SOS checkpoint by inhibiting the polymerization of FtsZ, a protein essential for cell division [8,9]. In spore-forming bacteria, including *Clostridioides* and *Bacillus* species, regulatory systems, including diadenylate cyclases (DACs), ensure genome integrity and sporulation [10,11]. The initiation of sporulation is tightly regulated by Spo0A [12]. In pathogenic species, this control is even more precise. In *Bacillus anthracis*, Spo0E-like proteins act as checkpoints that prevent sporulation during an active infection, promoting virulence [13]. In *Clostridioides difficile*, LexA controls the SOS response as well as processes related to virulence and motility [14].

The ability of spores of the genus *Bacillus* and *Clostridioides* to efficiently return to vegetative growth and propagate depends on their genomic integrity [1,4]. During spore germination and outgrowth, the activation of aerobic metabolism and hydration of the bacterial spore core can elicit the production of reactive oxygen species (ROS), which can damage DNA [1,15]. Therefore, DNA damage repair is necessary during this stage for efficient spore germination/outgrowth [6]. Previous studies have shown that ROS-elicited DNA lesions slow the progression of *B. subtilis* spore outgrowth [15]. These oxidative lesions, which include AP sites, DNA strand breaks, and oxidized bases, such as 8-OxoG, are mainly processed by the base excision repair (BER) pathway [7]. This repair system, which employs specific DNA glycosylases to process distinct chemically altered nucleobases, requires the ineludible contribution of AP-endonucleases (APEs) [7]. *B. subtilis* possesses three main APEs, namely, Nfo, ExoA, and Nth [7,16,17]. Nfo and ExoA counteract the effects of ROS by scavenging oxidative DNA lesions during spore germination/outgrowth [18]. Accordingly, *B. subtilis* spores lacking these AP-endonucleases (∆*nfo* ∆*exoA* strain) exhibit delayed germination/outgrowth [18]. *B. subtilis* possesses proteins with checkpoint functions, which regulate cell division in response to DNA damage including the DNA damage-scanning protein DisA [1,7]. DisA functions during stage *t*_2_ of the sporulation process to ensure that a chromosome copy is free of damage and segregates to the forespore [19]. Recent reports have shown that the checkpoint functions of DisA operate during the return of *B. subtilis* spores to vegetative growth [15,20]. Disruption of *disA* alleviated the delayed outgrowth exhibited by ∆*nfo* ∆*exoA* spores, thus unveiling a checkpoint role for DisA in this developmental stage [15]. In addition to Nfo and ExoA, *B. subtilis* possesses the APE Nth, which displays DNA glycosylase activity and can remove 8-OxoG lesions [21,22]. The disruption of the Nth encoding gene increased the susceptibility to H_2_O_2_ of the ∆*nfo* ∆*exoA* vegetative cells [17].

As noted above, the lack of Nfo and ExoA delays spore germination/outgrowth, and this phenotype is suppressed by the disruption of *disA* [15]. In this work, we report that DisA-mediated suppression of the delayed spore outgrowth displayed by cells deficient in Nfo and ExoA requires a functional *nth* gene, suggesting that other checkpoint pathways still occur during the outgrowth stage. Here, we analyzed (i) the germination/outgrowth properties, (ii) the chromosome replication status (iii) as well as the repair kinetics of oxidative DNA damage repair during the germination and outgrowth process. Overall, our results provide evidence indicating that in addition to DisA, outgrowing *B. subtilis* spores rely on alternative checkpoint mechanisms that are activated by ROS-promoted genetic lesions to ensure the successful return to vegetative growth for the spores.

## 2. Materials and Methods

### 2.1. Strain and Culture Conditions

The *B. subtilis* strains used in this study were derived from strain PS832, a prototrophic derivative of strain 168 [23], and are listed in Table 1. Liquid cultures of *B. subtilis* were grown routinely in Luria Bertani (LB) medium [24]. When required, erythromycin (Ery; 5 μg mL^−1^), chloramphenicol (Cm; 5 μg mL^−1^), tetracycline (Tet; 10 μg mL^−1^), or neomycin (Neo; 10 μg mL^−1^) were added to the media. Solid media were obtained by adding bacteriology grade agar (15 g L^−1^) to the liquid media. Liquid cultures were incubated at 37 °C with vigorous aeration. Cultures on solid media were incubated at 37 °C in the dark. Spores of all strains were prepared at 37 °C on Difco Sporulation Medium (DSM) [25] agar plates without antibiotics, harvested and purified by water washing, and stored as described previously [26]. All dormant spore preparations used in this work were free (≥98%) of growing cells, germinated spores, and cell debris, as determined by phase-contrast microscopy. Spores were generally used at an optical density at 600 nm (OD600) of 0.5 corresponding to 0.75 × 10^8^ viable spores mL^−1^ [20].

To generate the strain *B. subtilis* PERM1378 (Δ*nth* Δ*nfo* Δ*exoA* Δ*disA*), competent cells of the strain *B. subtilis* strain PERM769 Δ*nth* Δ*nfo* Δ*exoA* were transformed with the plasmid pPERM1372. The correct disruption of *disA* was confirmed by PCR using specific oligonucleotide primers.

### 2.2. Determination of Spore Germination and Outgrowth

Spore germination and outgrowth were performed in 2 × Schaeffer′s glucose (2 × SG) liquid medium [26] supplemented with 10 mM L-alanine. Spores in water were first heat shocked for 30 min at 70 °C, cooled on ice, and inoculated into a germination medium at 37 °C to obtain an initial OD_600_ of ~0.5 [20]. Where indicated, 0.5 mM hydrogen peroxide (H_2_O_2_) (Sigma-Aldrich, St. Louis, MO, USA) or 2 mM methyl methanesulfonate (MMS) (Sigma-Aldrich), equivalent to a 30% lethal dose of each drug [20], were added ~15 min after the germination onset. The concentrations of these agents were used in the experiments of spore germination/outgrowth and the determination of mutation frequencies to Rif^R^ for the WT and mutant strains. The OD_600_ of the cultures was monitored with an Ultrospec 2000 spectrophotometer (Pharmacia, Manassas Park, VA, USA), and the values were plotted as a fraction of the initial OD_600_ (OD_600_ at time t/initial OD_600_) versus time [20]. In all cases, the germination/outgrowth curves were performed with three different spore preparations and plotted as the average of the three replicates ± standard deviations.

### 2.3. Analysis of Spontaneous and H_2_O_2_- or MMS-Induced Mutation Frequencies

Spontaneous or induced mutations to rifampin resistance (Rif^R^) in outgrown spore cultures were determined as follows. Spores were germinated into flasks containing 2 × SG medium supplemented with 10 mM L-alanine. Fifteen minutes after germination initiation, each culture was divided in half, and the two halves were transferred to different flasks. While one of the cultures was left untreated, H_2_O_2_ or MMS as DNA-damaging agents were added to final concentrations of 0.5 mM and 2 mM, respectively. Aliquots removed from the control and treated cultures 180 min after the inoculation of spores into germination medium were spread on six LB medium plates containing 10 µg/mL rifampicin, and Rif^R^ colonies were counted after 1 day of incubation at 37 °C. The number of cells used to calculate the mutation frequency to Rif^R^ was determined by spreading aliquots of appropriate dilutions on LB medium plates without rifampin and incubating the plates for 24 to 48 h at 37 °C. These experiments were repeated at least three times.

### 2.4. Fluorescence Microscopy

Cell morphology, nucleoid structure, and DisA-Gfp foci synthesis were analyzed by epifluorescence microscopy. To this end, a previously reported protocol [20] was implemented with the following modifications. Briefly, samples (1 mL) of cultures from germinated spores were removed at various times, centrifuged (15,000× *g* [20 °C]), and mixed with 0.1 mL of fixative solution (3% [vol/vol] paraformaldehyde and 5% glutaraldehyde in HEPES buffered saline [273 mM NaCl, 9.9 mM KCl, 1.27 mM Na_2_HPO_4_·2H_2_O, 11.1 mM dextrose, 42 mM HEPES [pH 7]). After 30 min at room temperature, fixation was continued on ice for 50 min. The samples were washed twice by centrifugation with PBS and suspended in 100 µL of GTE (5 mM glucose, 25 mM Tris-HCl, 10 mM EDTA [pH 8.0]). Aliquots (10 µL) of this suspension were mixed with 5 µL of 2 µg/mL 4′,6′-diamino-2-phenylindole (DAPI) in water to stain DNA and were kept at room temperature for 30 min. Where indicated, cells were also suspended in 100 µL of 10 µg/mL FM4-64 (Invitrogen, Waltham, MA, USA) and kept at room temperature for 1 h. For microscopy, cell samples were prepared as previously described [27]. Fluorescence microscopy was performed with a ZEISS Axioscope A1 microscope (ZEISS, Oberkochen, Germany) equipped with an AxioCam ICc1 camera microscope (ZEISS, Oberkochen, Germany). Images were acquired with the AxioVision V 4.8.2 software and adjusted only for brightness and contrast.

Exposure times were typically 0.2 s for DAPI, 0.5 s for FM4-64, and 0.5 s for DisA-Gfp. Excitation and emission wavelengths employed were 350 and 470 nm for DAPI, 506 and 750 nm for FM4-64, and 475 and 508 nm for DisA-Gfp, respectively.

### 2.5. Isolation and Quantitation of Chromosomal DNA

To quantify the chromosomal DNA from the germinated and dormant spores, chromosomal DNA was isolated following the protocol reported previously [20]. Briefly, aliquots (3 mL; 1.5 × 10^8^ viable spores mL^−1^) of WT, ∆*nth* ∆*nfo* ∆*exoA*, and ∆*nth* ∆*nfo* ∆*exoA* ∆*disA* dormant spores that had germinated for 90 min in 2 × SG medium were collected by centrifugation (14,000× *g* for 1 min). The pellet of cells was washed twice with 1 mL of lysis buffer (50 mM EDTA, 100 mM NaCl [pH 7.5]), suspended in 0.3 mL of the same buffer, and subsequently processed to isolate the RNA-free chromosomal DNA from the fraction that was directly susceptible to lysozyme degradation, as previously described [15]. The fraction of lysozyme-resistant cells was pelleted by centrifugation. This pellet was subjected to spore coat removal [27], washed five times with STE buffer (150 mM NaCl, 10 mM Tris-HCl [pH 8], 10 mM EDTA), and processed for chromosomal DNA isolation [15]. After RNAse treatment, the chromosomal DNA isolated from both fractions was quantified by UV spectrophotometry [28]. The DNA values from both fractions were combined to obtain the total DNA content.

### 2.6. Detection of Oxidative DNA Damage

Oxidative damage in the chromosomal DNA of the strains of interest was performed by alkaline gel electrophoresis employing a previously described protocol [15]. Briefly, samples of chromosomal DNA isolated from the dormant and germinated spores of each strain were left untreated or incubated with 14 units of formamidopyrimidine-DNA glycosylase (Fpg, New England BioLabs, Ipswich, MA, USA), which cleaves DNA at the 8-oxoguanine (8-OxoG) residues, according to the manufacturer’s instructions. Enzyme reactions were carried out for 1 h, and reaction mixtures containing 3 or 5 µg of DNA were then electrophoresed on a 1% alkaline agarose gel, which was then stained with ethidium bromide, as described previously [28].

### 2.7. Statistical Analysis

Statistical differences in the mutagenesis rate between the untreated and treated strains with the damaging agents (H_2_O_2_ and MMS) were calculated using a Mann–Whitney U test with a 95% confidence level (*p* < 0.05). All tests were performed using a licensed version of the Minitab 18 software.

## 3. Results

### 3.1. Analysis of Germination and Outgrowth of B. subtilis Spores Deficient for APEs and DisA

**Figure 1:**
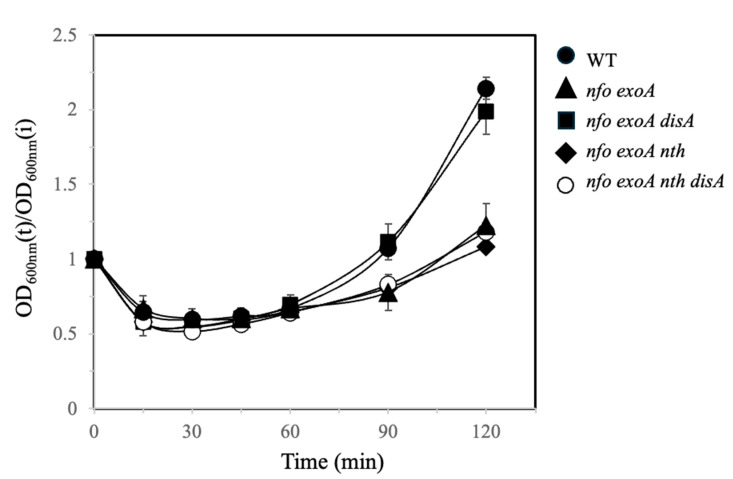
Germination/outgrowth kinetics of different strains of *B. subtilis* spores. Dormant spores of *wt* (●), *nfo exoA* (▲), *nfo exoA nth disA* (◼), *nfo exoA nth* (◆), and *nfo exoA nth disA* (◯) strains were heat activated and germinated on 2 × SG medium. Germination and outgrowth were monitored by measuring the OD_600_nm of the cultures as described in Section 2. The kinetics were performed with three different spore preparations and plotted as described in Section 2.

AP-endonucleases are necessary to repair AP sites generated from oxidative lesions [4,7,29]. A previous study revealed that the lack of Nfo and ExoA caused a delayed spore outgrowth in *B. subtilis*, presumably due to the accumulation of ROS-promoted DNA damage [18]. Interestingly, in addition to Nfo and ExoA, *B. subtilis* counts with Nth, an enzyme capable of processing APs and 8-OxoG lesions [17,21,22,30]. Therefore, we sought to investigate the germination and outgrowth properties of spores with deficiencies in Nfo and ExoA that also lacked Nth. Results showed that spores with deficiencies in Nfo, ExoA, and Nth exhibited a delayed outgrowth in comparison with spores of the WT strain (Figure 1). As previously shown, genetic disruption of *disA* in the Δ*nfo* Δ*exoA* spores was shown to alleviate the delayed return of these AP deficient spores to vegetative growth (Figure 1, [15]); this observation prompted us to generate spores with disrupted *disA* in the Δ*nfo* Δ*exoA* Δ*nth* genetic background. However, spores from the APEs/DisA-deficient strain did not restore their germination/outgrowth to the levels exhibited by the WT spores (Figure 1). These results suggest that genetic lesions that are not repaired by Nth could trigger a DisA-independent checkpoint event that slows spore germination/outgrowth.

### 3.2. Effect of H_2_O_2_ and MMS During Germination/Outgrowth of B. subtilis Spores Deficient in AP-Endonucleases and DisA

**Figure 2:**
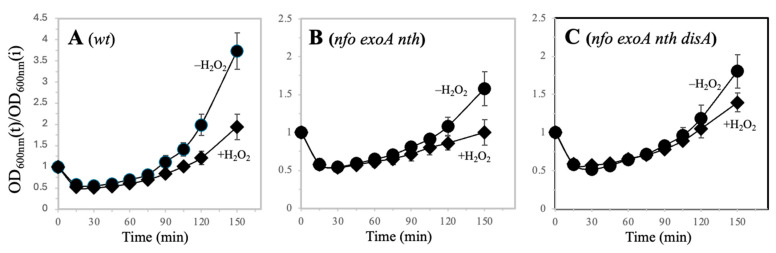
Germination/outgrowth kinetics of different strains of *B. subtilis* spores. Dormant spores of strains (**A**) *wt* (●) and *wt* H_2_O_2_ (◆), (**B**) *nfo exoA nth* (●) and *nfo exoA nth* H_2_O_2_ (◆), (**C**) *nfo exoA nth disA* (●) and *nfo exoA nth disA* H_2_O_2_ (◆) were heat shocked and germinated by the addition of L-alanine. Fifteen minutes after germination was initiated, H_2_O_2_ was added to the cultures to a final concentration of 0.5 mM. Germination and outgrowth were monitored by measuring the OD600nm of the cultures as described in Section 2. The kinetics were performed with three different spore preparations and plotted as described in Section 2.

The delayed outgrowth of spores deficient for Nfo and ExoA has been proposed to be triggered by the accumulation of genetic lesions of oxidative nature and the activation of DNA checkpoints [15,18]. Next, we investigated whether the ROS promoter agent H_2_O_2_ influenced the germination and outgrowth processes of spores deficient for Nfo, ExoA, and Nth as a function of DisA. The results showed that H2O2 slowed down the outgrowth of the wild-type spores, and in the ∆APEs and ∆APEs ∆*disA* spores, this oxidizing agent exacerbated the delay in outgrowth even further (Figure 2A–C).

**Figure 3:**
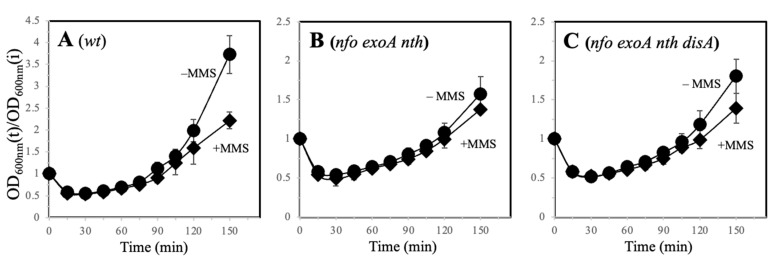
Germination/outgrowth kinetics of different strains of *B. subtilis* spores. Dormant spores of strains (**A**) *wt* (●) and *wt* MMS (◆), (**B**) *nfo exoA nth* (●) and *nfo exoA nth* MMS (◆), (**C**) *nfo exoA nth disA* (●) and *nfo exoA nth disA* MMS (◆) were heat shocked and germinated by the addition of L-alanine. Fifteen minutes after germination was initiated, MMS was added to the cultures to a final concentration of 2 mM. Germination and outgrowth were monitored by measuring the OD600nm of the cultures as described in Section 2. The kinetics were performed with three different spore preparations and plotted as described in Section 2.

The DNA-damaging agent MMS promotes base mispairings and generates repair intermediates that affect replication and transcription [31]. While MMS affected the WT spores during vegetative growth, this alkylating agent did not impact the germination/outgrowth of the WT, Δ*nfo* Δ*exoA* Δ*nth*, and Δ*nfo* Δ*exoA* Δ*disA B. subtilis* spores (Figure 3A–C). Taken together, these results suggest that during outgrowth, the AP endonucleases prevent the cytotoxic effects of oxidative lesions and that additional repair pathways can process DNA base alkylation in spores deficient for Nfo, ExoA, and Nth.

### 3.3. Spontaneous, H_2_O_2_- and MMS-Induced Mutagenesis During B. subtilis Spore Outgrowth

**Figure 4:**
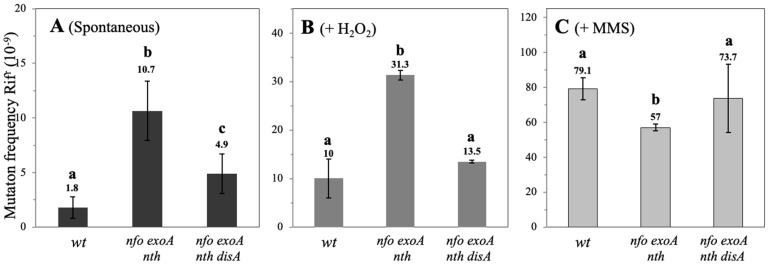
Frequency of spontaneous and induced mutation during germination/outgrowth of *B. subtilis* spores. Spore suspensions of the indicated strains were activated by heat shock and then supplemented with L-alanine to induce germination. In (**A**) 180 min after initiation of gemination, the mutation frequency to Rif^r^ was determined as described in Section 2. In (**B**,**C**), 15 min after germination initiation, H_2_O_2_ and MMS damage agents were added, respectively, and subsequently, at 180 min of incubation, the Rif^r^ mutation frequency was determined as described in Section 2. Values represent the mean of data collected from three independent experiments, and error bars represent the standard deviation. Letters above bars indicate statistical differences found by a Mann–Whitney U test (*p* < 0.001).

To better assess whether the delayed outgrowth phenotype exhibited by APE-deficient spores obeys to DNA damage, we determined the spontaneous and induced mutagenesis in outgrown *B. subtilis* spores proficient or deficient for APEs. Compared with WT spores, spores from the Δ*nfo* Δ*exoA* Δ*nth* strain exhibited 6-fold increased levels of spontaneous Rif^R^ mutagenesis. Of note, disruption of *disA* in the Δ*nfo* Δ*exoA* Δ*nth* genetic background generated outgrown spores that were 1.8 times more mutagenic than spores of the WT strain (Figure 4A) but showed a decrease of ~50% in the frequency of spontaneous mutation compared with the strain lacking Nfo, ExoA, and Nth (Figure 4A). The oxidizing agent H_2_O_2_ increased mutagenesis by ~12.5-, ~17-, and ~7.5-fold in the outgrown WT, Nfo/ExoA/Nth, and Nfo/ExoA/Nth/DisA-deficient spores, respectively (Figure 4B). Notably, disruption of *disA* resulted in decreases in the mutagenesis levels in the outgrowth of the triple *nfo*, *exoA*, *nth* mutant by about 50% (Figure 4B). Altogether, these results suggest that DisA promotes error-prone repair events in outgrowing cells deficient for the main APEs.

The alkylating agent MMS promoted a ~44-fold increase in the levels of Rif^R^ mutagenesis in outgrown spores of the WT strain (Figure 4C). While these levels decreased ~1.4-fold in outgrown spores deficient for the three APEs; they remained unaffected in outgrown spores of the quadruple Δ*nfo* Δ*exoA* Δ*nth* Δ*disA* strain (Figure 4C). In conjunction, these results suggest that during spore outgrowth, Nfo, ExoA and Nth prevent ROS-promoted mutagenesis and that alkylated bases are repaired through a pathway independent of these APEs. Furthermore, they suggest that DisA can elicit an error-prone repair pathway in the presence of oxidative lesions that compromise genome fidelity.

### 3.4. Determination of 8-OxoG and FapyGua Lesions by Alkaline DNA Electrophoresis (AGE)

**Figure 5:**
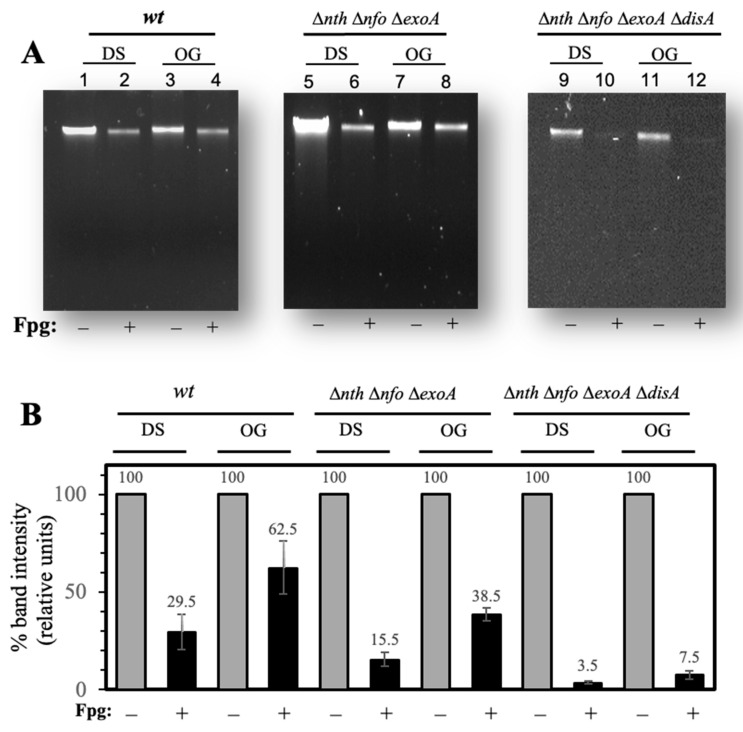
Determination of oxidative DNA lesions by alkaline gel electrophoresis (AGE). (**A**) Genomic DNA samples (3 µg) from dormant (DS) and outgrowing (OG) spores of *wt* (Left panel; lanes 1–4), *nfo exoA nth* (Middle panel; lanes 5–8), and *nfo exoA nth disA* (Right panel; lanes 9–12), were incubated without Fpg treatment (lanes 1, 3, 5, 7, 9, 11) or treated with 14 units of Fpg (lanes 2, 4, 6, 8, 10, 12). The reaction products were separated on 1% alkaline agarose gels and stained with ethidium bromide as described in Section 2. The data shown are representative of the results of two independent experiments. (**B**) Quantification of chromosomal DNA degradation from experiments shown in Figure 5A, were determined by densitometry using ImageJ 1.47n software. The analyses were performed with two alkaline gels (with different batches of outgrown DNA spores). Values represent the average of the two experiments ± standard deviations.

Employing alkaline gel electrophoresis (AGE), we next determined the type of ROS-promoted DNA lesions delaying outgrowth and leading to mutagenesis in strains deficient for APEs and DisA. Purified chromosomal DNA from dormant (DS) and outgrowing (OG) spores were treated with the Fpg, a glycosyl hydrolase that operates on 8-OxoG and 2,6-diamino-4-hydroxy-5-formamidopyrimidine (FapyGua) lesions [32,33]. The DNA products resulting from the enzymatic attack of 8-OxoG were separated by AGE [28]. A smear or decrease in the high molecular weight DNA fragment indicated a greater number of oxidative lesions in the DNA sample. Furthermore, we quantified the intensity of the chromosomal band that remained following Fpg treatment in reference to an untreated control as a measure of the content of 8-OxoG lesions by densitometry (Figure 5B). The results showed the presence of 8-OxoGs in chromosomal DNA from the WT spores; however, such lesions were significantly eliminated during outgrowth (Figure 5A left panel and Figure 5B). In contrast, a significant proportion of the 8-OxoG lesions detected in the chromosomal DNA of spores deficient for the three major APEs were displayed during outgrowth (Figure 5A middle panel and Figure 5B). Notably, the genetic inactivation of DisA in the strain deficient for APEs generated spores that retained a greater number of 8-OxoG lesions, as revealed by the full degradation of the chromosomal DNA by Fpg (Figure 5A right panel and Figure 5B), during outgrowth. Altogether, these results support the notion that APES and DisA are key factors that counteract the detrimental effects of ROS-promoted genetic lesions during spore outgrowth.

### 3.5. Chromosome Replication Is Delayed During Outgrowth of B. subtilis Spores Deficient for APEs and DisA

**Figure 6:**
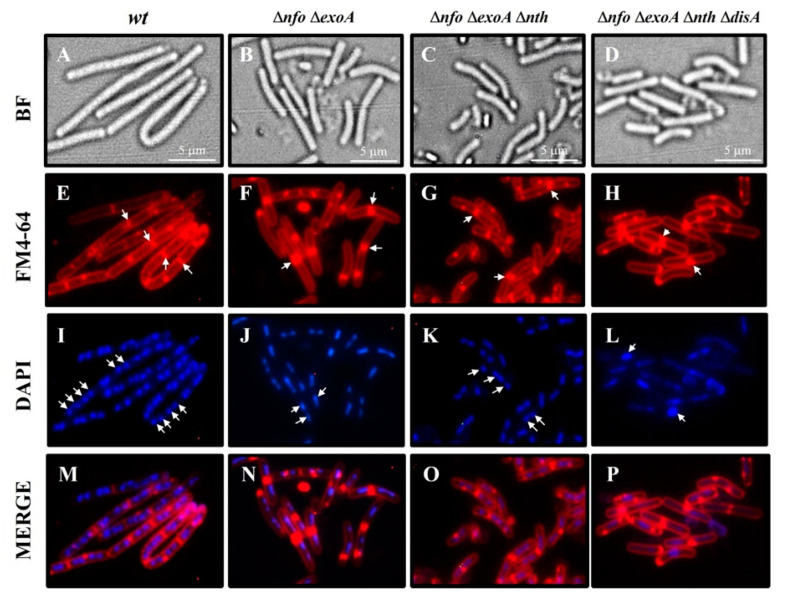
Microscopic analysis of chromosome replication status 90 min after the germination onset of spores with different phenotypes. Spores from *wt*, *nfo exoA*, *nfo exoA nth*, and *nfo exoA nth disA* strains were heat-shocked and germinated in 2 × SG medium supplemented with L-alanine. Cell samples were collected 90 min after the onset of spore germination, fixed, and stained as described in Section 2. The cells were analyzed by bright-field (BF) and fluorescence microscopy (FM4-64 and DAPI staining). Scale bar, 5 μm. (**A**–**D**) Bright field; (**E**–**H**) FM4-64 staining; (**I**–**L**) DAPI staining; (**M**–**P**); overlain images of FM4-64 and DAPI are depicted as MERGE. Arrowheads indicate septum stained with FM4-64 and chromosomes stained with DAPI, respectively.

We used epifluorescence microscopy to investigate whether the delayed return to vegetative growth in spores deficient for the major APEs and differing in DisA proficiency was associated with defects in chromosomal replication. The results revealed that spores of the *wt* strain had undergone several division cycles as well as multiple rounds of chromosomal replication (Figure 6E,I) by 90 min after the onset of germination. In contrast, spores of the mutant strains exhibited a delay in both cell division and the replication of their chromosomes (Figure 6J–L) with respect to the WT strain. Specifically, spores deficient for *nfo* and *exoA* appeared to have divided and replicated their DNA once (Figure 6N,J). Disruption of *nth* in the ∆*nfo* ∆*exoA* background generated outgrowing spores with more septa (Figure 6G) and more chromosomes (Figure 6K) relative to the spores of the Nfo/ExoA-deficient strain. Strikingly, disruption of DisA in spores deficient for the three APEs generated, during outgrowth, cells that lacked a septum (Figure 6H), and most had a single chromosome (Figure 6L). Notably, during outgrowth, some chromosomes of the ∆APEs ∆*disA* strain exhibited a larger mass (Figure 6L) than those observed in the WT and APEs deficient strains, suggesting that the APEs/DisA-deficient spores replicate their chromosomes but are unable to segregate them in this developmental stage.

### 3.6. DisA Foci Synthesis in Outgrowing B. subtilis Spores Deficient and Proficient for APEs

**Figure 7:**
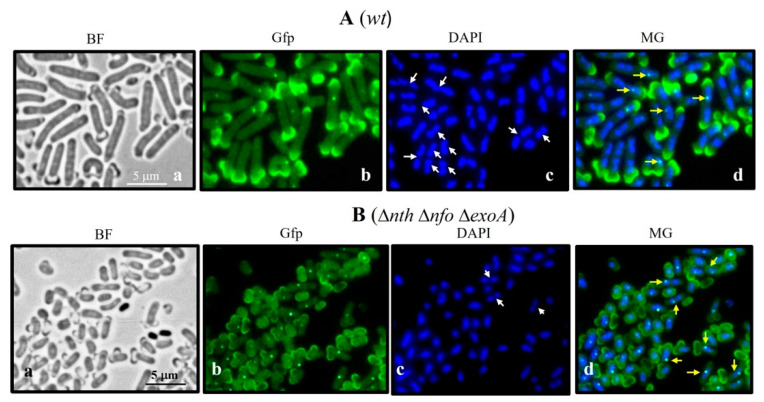
Microscopic analysis of the DisA-Gfp foci synthesis. Outgrown spores of the strains *wt* (**A**) or *nfo exoA nth* (**B**) collected 90 min after the germination onset were fixed and stained as described in Section 2. The cells were analyzed by bright field (BF) and fluorescence microscopy (DAPI and Gfp staining). Scale bar, 5 μm. In (**A**,**B**); a–d: BF, bright field; Gfp, DisA-Gfp fluorescence; DAPI, chromosomal fluorescence; MG, overlain images of Gfp and DAPI. White arrowheads show replicated chromosomes; yellow arrowheads indicate DisA-Gfp foci.

The expression of *disA* takes place during spore outgrowth, and its encoded product generates multimeric foci that colocalize with the chromosome [15]. We inspected the dynamics of DisA-Gfp foci synthesis in outgrown spores deficient and proficient for Nfo, ExoA, and Nth by fluorescence microscopy. Our results showed that in reference to the outgrown *wt* spores, spores deficient for APEs exhibited a delayed outgrowth process and replication of their chromosomes (Figure 6 and Figure 7). However, formation of the DisA-Gfp foci was observed in both strains (Figure 7). These results, together, with those shown in Figure 1 and Figure 6, strongly suggest that in Nfo/ExoA/Nth-deficient spores, DisA-dependent and independent checkpoints are activated to delay the first round of chromosomal replication.

## 4. Discussion

In this work, we investigated how the loss of the APEs and the consequent accumulation of oxidative DNA damage impacted the germination/outgrowth of *B. subtilis* spores differing in the checkpoint factor DisA. Results from the germination and outgrowth kinetics of spores as well as the determination of mutagenesis and repair of oxidative DNA damage of APEs/DisA-deficient spores unveiled the existence of DisA-dependent and independent DNA-damage checkpoints during the return to vegetative growth.

While disruption of *disA* suppresses the slow outgrowth phenotype of spores with deficiencies in Nfo and ExoA [15], this outcome was not observed in spores that lacked Nfo, ExoA, and Nth (Figure 1). These results suggest that DNA lesions that are left unprocessed by Nth activate DisA-independent checkpoint(s) during germination/outgrowth. Nth, together with MutY, belongs to a family of type III endonucleases that contain a Fe-S cluster and a helix-turn-helix domain to bind DNA [34,35,36]. The Nth repair protein operates over AP sites but can also act as a DNA glycosylase capable of hydrolyzing 8-OxoG lesions in DNA [22,34]. A previous report revealed that the processing of AP sites through Nth, Nfo, and ExoA have a divergent impact on growth- and stationary phase-associated mutagenesis in *B. subtilis* [17]. As shown in this work, Nth together with Nfo and ExoA play an antimutagenic role by preventing spontaneous and ROS-promoted mutagenesis (Figure 4). Furthermore, our results revealed that in addition to preventing the mutagenic impact of AP sites, Nth can also counteract the genetic damage promoted by 8-OxoG in outgrowing spores (Figure 5). Based on this observation, we propose that the repair properties of Nth are important for the initial stages of chromosomal replication, specifically during the DnaD-dependent untwisting of the replication origin [21]. Interestingly, *dnaD* and *nth* are arranged in the same operon in the chromosome of *B. subtilis* and could be expressed during spore/germination outgrowth [21,37].

The checkpoint function of DisA is activated by bulky DNA lesions promoted by nalidixic acid (Nal) and mitomycin C (MC) during the initial stages of the sporulation process [38]. In this developmental stage, DisA scans the chromosomal copies to assess their integrity before segregating one of the copies to the forespore compartment [19,38]. Here, we found that DisA participates in processing the ROS-promoted lesion 8-OxoG, or its repair intermediates, as its deficiency negatively impacted the processing of these genetic insults during the outgrowth of spores lacking the major APEs. Notably, as revealed by the determination of spontaneous and ROS-promoted mutagenesis, these DisA-dependent repair transactions can involve error-prone DNA synthesis (Figure 4). Indeed, a previous report showed that DisA promotes error-prone repair in outgrowing spores deficient in the transcription coupling repair factor Mfd [20]. These observations suggest that YqjH/YqjW (PolY1/PolY2) are active in *B. subtilis* and agree with previous reports showing their effects on cells experiencing DNA damage [39]. Furthermore, these replicases play key roles in an alternative excision repair pathway (AER) that protects sporulating cells from UV-C light as well as in modulating stress-associated mutagenesis (SAM) [40].

Our results showed that outgrowing spores deficient for APEs and DisA were maladapted in the response to DNA oxidants; however, these experiments also revealed that spores of these mutant strains can prevent the mutagenic effects promoted by the DNA alkylating agent MMS [31]. We postulate that repair pathway(s) other than from BER efficiently process base methylation during outgrowth in these mutant spores. Perhaps the DNA damage induced by MMS can be repaired by the NER system, whose encoding genes *uvrA*, *uvrB*, and *uvrC* are under control of the SOS regulon in *B. subtilis* [4]. In support of this notion, a previous report revealed that this transcriptional circuit is gratuitously induced during spore/germination outgrowth [20]. However, additional repair systems, including the DNA glycosylase Aag as well as the methyl transferases AdaA and AdaB, can be involved in counteracting the base damage promoted by MMS in this developmental stage [7,27,41]. Our microscopy analyses suggest that AP sites or their repair intermediates elicited a delay in chromosome replication during the outgrowth of spores deficient for APEs and DisA (Figure 6 and Figure 7). The presence of these genotoxic lesions can initially preclude the efficient replication of the outgrowing spore’s chromosome through the main replicative polymerase PolC. We speculate that alternative DNA replicases, which tolerate DNA damage, are active during spore outgrowth. Indeed, genetic lesions, including AP sites that arrest the progress of the replisome, can elicit the recruitment of DnaE and TLS polymerases that catalyze the synthesis of short stretches of DNA and prevent the cytotoxic consequences of stalled replication forks [42,43,44]. In *B. subtilis*, a recent report suggested that PolY1 (YqjH) and the β clamp (DnaN) form a complex, and that this complex is a constitutive component of the replisome that facilitates progression at sites of DNA damage and prevents the generation of cytotoxic strand breaks [45,46]. On the other hand, stalled replication forks can activate the SOS response [7] and induce the expression of *polY2*, encoding a replicase that bypasses AP sites by TLS synthesis [43]. Furthermore, it is also possible that, as has previously been shown [43], the replication stress caused by AP sites can be counteracted by the recombination machinery during spore germination/outgrowth.

Here, we found that a functional DisA-Gfp foci could still be formed in outgrown spores lacking Nfo, ExoA, and Nth (Figure 7), suggesting that the delayed replication in this genetic context is activated by DisA-dependent and independent checkpoints. Indeed, the genetic disabling of *disA* did not suppress the slowed replication phenotype (Figure 1 and Figure 5), thus providing support for this hypothesis.

The return to vegetative growth by spores requires a high rate of transcription as a large number of proteins are needed to activate metabolism and generate nucleotide precursors for the first round of chromosome replication [1]. During this stage, spore-packed proteins play a pivotal role in eliminating DNA lesions that can lead to pauses in RNA polymerase and create potential blocks to the DNA replication machinery [1,47]; these events are processed by Mfd [20]. Therefore, delays in chromosomal replication may not only obeys to inefficient DNA synthesis, but also to Mfd-promoted checkpoints activated by oxidative DNA lesions including 8-OxoG, AP sites, and single-strand breaks occurring in the chromosomal template strand. A previous study provides supports for this hypothesis, indicating that Mfd and DisA act in a coordinated manner to process genetic damage that interferes with the first rounds of transcription and replication taking place during spore outgrowth [20].

In summary, during spore germination and outgrowth, (i) Nth plays a key role in eliminating genetic lesions of oxidative nature that can interfere with replication, DNA repair and recombination, and (ii) the accumulation of AP sites, 8-OxoGs, or their repair intermediates activate DisA-independent mechanisms that promote genetic diversity, impacting cell fitness and thus leading to a successful return of the spores to vegetative growth.

## Figures and Tables

**Table 1:** Bacterial strains used in this study.

Strain	Genotype and Description	Source or Reference
**Strains** (*B. subtilis*)		
PS832	Wild type, *trp*^+^, revertant of strain 168	[23]
PERM454	∆*nfo*::neo ∆*exoA*::tet Neo^r^ Tet^r^	[18]
PERM769	∆*nfo*::neo ∆*exoA*::tet ∆*nth*:ery Neo^r^ Tet^r^ Ery^r^	[17]
PERM1378	∆*exoA*::tet ∆*nfo*::neo ∆*nth*::ery ∆*disA*::cm Neo^r^ Tet^r^ Ery^r^ Cm^r^	This study
PERM1008	PS832 *disA-Gfp*::ery Ery^R^	This study
PERM1685	∆*nfo*::neo ∆*exoA*::tet ∆*nth*:cm *disA-Gfp*::ery Neo^r^ Tet^r^ Cm^R^ Ery^r^	This study
**Plasmids**		
pPERM1372	pMutin4cat containing an internal region (307 bp) of *disA*; Cm^r^	[20]

## Data Availability

The original contributions presented in this study are included in the article. Further inquiries can be directed to the corresponding author.
